# The effect of a newly developed mini-light-emitting diode catheter for interstitial photodynamic therapy in pancreatic cancer xenografts

**DOI:** 10.1186/s12967-021-02900-8

**Published:** 2021-06-07

**Authors:** So-Young Kim, Eun A. Cho, Sang Mun Bae, Sang-Yeob Kim, Do Hyun Park

**Affiliations:** 1 Asan Medical Center, Asan Institute for Life Sciences, University of Ulsan College of Medicine, Seoul, Korea; 2 Department of Medical Science, Asan Medical Center, University of Ulsan College of Medicine, Seoul, Korea; 3 Asan Medical Center, Asan Institute for Life Sciences, University of Ulsan College of Medicine, 88, Olympic-ro 43-gil, Songpa-gu, Seoul, 05505 Korea; 4 Division of Gastroenterology, Department of Internal Medicine, Asan Medical Center, University of Ulsan College of Medicine, 88, Olympic-ro 43-gil, Songpa-gu, Seoul, 05505 Korea

To the Editor

The incidence of and mortality due to pancreatic cancer, with a low rate of response to chemotherapy or radiotherapy, are increasing worldwide [1–4]. Photodynamic therapy (PDT) has already been approved by the Food and Drug Administration and has been used in clinical trials for oncological treatment, including that for pancreatic cancer [5]. PDT is widely applied for promoting selective tumor necrosis or apoptosis using light after administering a photosensitizer [4]. Chemotherapy after PDT might occasionally lead to tumor downstaging, thereby allowing an attempt at surgical resection or R0 resection in patients with locally advanced pancreatic cancer [4]. For delivering light, the percutaneous approach might be uncomfortable for patients and requires passing the fiber laser until a long distance from pancreatic mass. Therefore, endoscopic ultrasound (EUS)-guided interstitial PDT (i-PDT) might be an optimal modality to deliver PDT to the pancreas mass [3, 4]. A recent consensus statement from an expert panel for PDT use in pancreatic cancer also recommended that 1) light delivery should be accomplished using EUS guidance, and 2) PDT can be used to downstage the pancreatic cancer before surgical resection [6].

However, a fiber laser used for i-PDT might be fragile and costlier and cause light loss during endoscopic procedures due to multiple flexions of the fiber laser that are required [3]. Furthermore, an unexpected small volume of necrosis might occur because a small amount of blood around the tip of the fiber laser might reduce the transmission of light energy into the target tissue [2–4].

Recent advances in mini light-emitting diodes (LEDs) may have made possible the development of a miniaturized catheter for i-PDT. We hypothesized that the newly developed mini-LED catheter might enhance light transmission with higher efficiency and lesser light energy loss than the conventional fiber laser in pancreatic cancer xenografts.

Human pancreatic cancer BxPC-3 cell lines were used to establish xenograft models in BALB/c nude mice (male, 8 weeks old, Charles River, Yokohama, Japan). Two widely available photosensitizers (Photofrin, Concordia Laboratories Inc, St Michael, Barbados and Chlorin e6, Ce6, Frontier Scientific, UT, USA) were used. Photofrin (5 mg/kg, porfimer sodium) was intravenously injected in mice having tumors larger than 8–10 mm. The mice were kept in dark conditions for 24 h, following which their tumors were treated with i-PDT through the conventional (5 mm-length conventional optical quartz diffuser, 630 nm, power density of 300 mW/ cm^2^, 100 J/cm^2^) or mini-LED catheter (5 mm-length diffuser, 630 nm, power density of 8.5 mW/cm^2^) in the Photofrin group. Intravenous administration of Ce6 (2.5 mg/kg, Frontier Scientific, UT, USA) and laser irradiation at 660 nm (5 mm-length conventional optic quartz diffuser, power density of 300 mW/cm^2^, 100 J/cm^2^) or min-LED catheter (5 mm-length diffuser, 650 nm, power density of 9.1 mW/cm^2^) were performed in the Ce6 group (Fig. 1A–D). In both groups, a dose de-escalating scheme was designed with a 100 J/cm^2^ and 50 J/cm^2^ in mini-LED based i-PDT for the evaluation of durable light intensity compared with conventional dose (100 J/cm^2^) of fiber laser based i-PDT. Mice were divided into five treatment groups: i) Photofrin or Ce6 + conventional i-PDT with 100 J/cm^2^; ii) Photofrin or Ce6 + mini-LED i-PDT with 100 J/cm^2^; iii) Photofrin or Ce6 + mini-LED i-PDT with with 50 J/cm^2^; iv) free Photofrin or Ce6 + mini-LED irradiation with 100 J/cm^2^; and v) Photofrin or Ce6 alone. The changes in volume and size of xenograft tumors were measured within 15 days after i-PDT, and histopathologic examination was performed at the same time. For immunohistochemical analysis, the fixed tumor tissues were embedded in paraffin, sectioned (4 μm) onto glass slides, and stained with anti-CD68 antibody (ab125212, Abcam). Slides were scanned using the PerkinElmer Vectra Polaris Automated Quantitative Pathology Imaging System (Akoya Biosciences, Marlborough, MA), and images were analyzed using the inForm software and TIBCO Spotfire (Perkin-Elmer, Waltham, MA).

Among the treatment groups, mini-LED based i-PDT with Photofrin and Ce6 (100 J/cm^2^) resulted in the smallest tumor volume after treatment, with a reduction by about 48% and 41%, respectively, compared to the no-treatment control group, followed by the conventional i-PDT (100 J/cm^2^) with Photofrin (about 21% reduction), and Ce6 groups (about 17% reduction) (Fig. 1D, E). Growth curves in the mini-LED based i-PDT (Photofrin, 100 J/cm^2^ and 50 J/cm^2^; Ce6, 100 J/cm^2^) and conventional PDT (Photofrin 100 J/cm^2^) groups were significantly different than those in untreated controls (Fig. 2A, B).

**Fig. 1:**
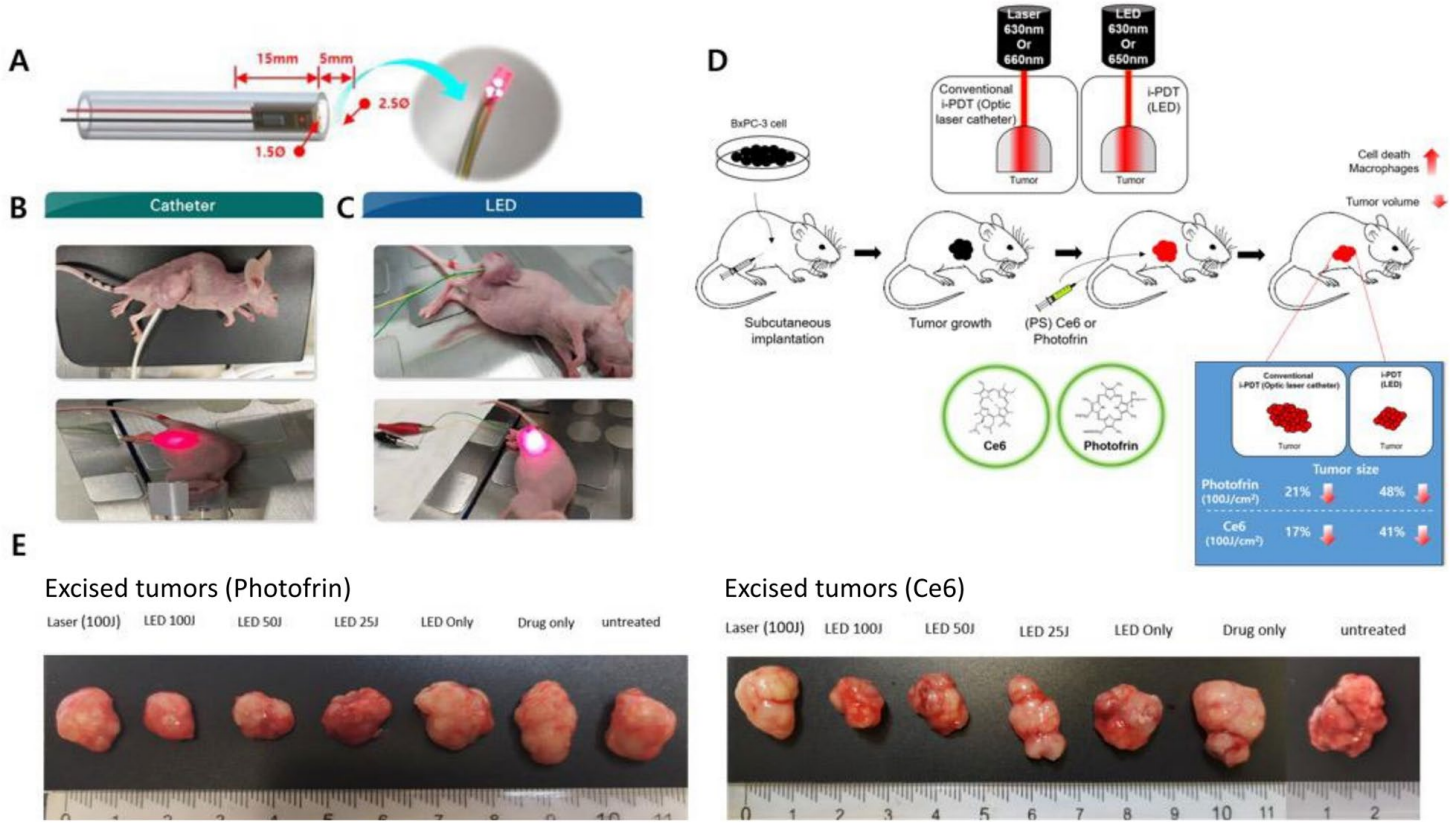
Schematic of the experimental study design. **A** LED catheter. Insertion of the FPCB (flexible printed circuit board) with a mini-LED bonded to the catheter (outer diameter 2.5 mm) and encapsulation with silicone. **B**, A conventional optic laser fiber catheter (RD; Medlight, Ecublens, Switzerland). **C**, A mini-LED catheter. After inserting into the animal model, the mini-LED was turned on by supplying power with the wire. **D** Follow-up of the experiment. **E** Comparison of the excised tumor after treatment (left, Photofrin; right, Ce6). Both LED-based i-PDT (100 J/cm2) with Photofrin and Ce6 had better suppression of tumor growth than in other groups. The excised tumor after LED-based i-PDT (25 J/cm2) is shown in this measurement as an addendum

After PDT, the recruitment of macrophages was assessed by histologic analysis of the CD68 immunohistochemical staining for the evaluation of i-PDT based dead cell removal [7, 8]. Tumors treated with mini-LED-based i-PDT with Photofrin (100 J/cm^2^) showed a significant increase in the recruitment of macrophages than in the untreated control group (Fig. 2C, D).

LEDs, unlike the conventional light sources, generate high levels of light with less heat; they have a compact structure and low cost and consume less electricity [9]. Mini-(100–200 μm) or micro-LEDs (less than 100 μm) might have a greater clinical impact on wearable or implantable medical devices in terms of small size and application across various dimensions (9). In this preliminary report, 3 mini-LED chips (2 with side and 1 with forward firing) a with 2.5-mm diameter and 5-mm diffuser length catheter were used for administering PDT in pancreatic cancer xenografts (Fig. 1A).

**Fig. 2:**
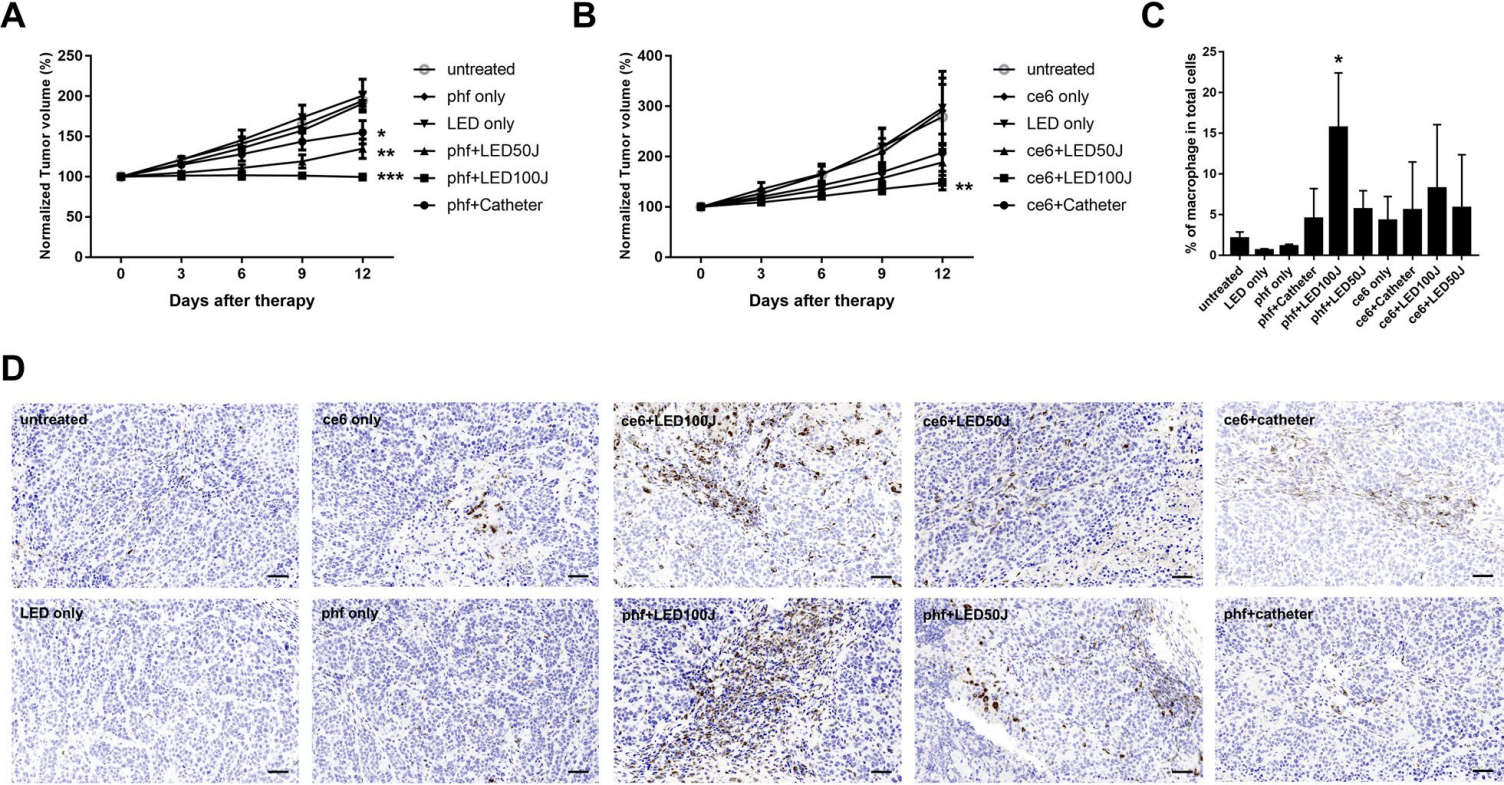
In vivo efficacy of interstitial-photodynamic therapy (i-PDT) in BxPC3 tumor-bearing mice within 15 days in different groups treated with Photofrin (Phf) and chlorin e6 (Ce6). **A**, Normalized tumor volumes in mice treated with Phf+Catheter, Phf+LED100J, Phf+LED50J, LED only, and Phf only and untreated mice (n = 4 per group). **B**, Normalized tumor volumes in mice treated with Ce6+Catheter, Ce6+LED100J, Ce6+LED50J, LED only, and Ce6 only and untreated mice (n = 4 per group). **C**, Quantitative analysis of CD68-positive cells after indicated treatments. **D**, Representative images of CD68 staining. Scale bars: 50 mm. Asterisks indicate p values for the comparison of each group of irradiated tumors or nonirradiated tumors by one-way ANOVA. *p< 0.05; **p<0.01; ***p<0.001

This miniaturized LED catheter might have a better clinical impact, with a twofold higher suppression of tumor growth and cost advantage compared with fiber laser. Considering the better suppression of the tumor growth in mini-LED-based i-PDT with lower power density and light dose compared with conventional i-PDT, mini- or micro-LED-based miniaturized catheters might be a suitable and promising downstaging option for i-PDT with impactful light delivery in patients with pancreatic cancer treated with neoadjuvant chemotherapy. Development of further miniaturized mini- or micro-LED catheters less than 1 mm in diameter and diffuser length of 1 cm for EUS-guided i-PDT is currently ongoing.

The mini-LED-based i-PDT with Photofrin and Ce6 showed promising antitumor effects in this pre-clinical study of xenografts of pancreatic cancer. Our data may provide preliminary evidence about the enormous potential of mini-LED-based i-PDT in patients with pancreatic cancer.

## Data Availability

The datasets used and/or analysed during the current study are available from the corresponding author on reasonable request.

## Abbreviations

PDT: Photodynamic therapy
EUS: Endoscopic ultrasound
LED: Light emitting diode
i-PDT: Interstitial PDT
Ce6: Chlorin e6
